# Effects of Mild Blast Traumatic Brain Injury on Cerebral Vascular, Histopathological, and Behavioral Outcomes in Rats

**DOI:** 10.1089/neu.2017.5256

**Published:** 2018-01-15

**Authors:** Uylissa A. Rodriguez, Yaping Zeng, Donald Deyo, Margaret A. Parsley, Bridget E. Hawkins, Donald S. Prough, Douglas S. DeWitt

**Affiliations:** ^1^ Cell Biology Graduate Program, Department of Neuroscience and Cell Biology, Department of Anesthesiology, University of Texas Medical Branch, Galveston, Texas.; ^2^ The Moody Project for Translational Traumatic Brain Injury Research, Charles R. Allen Research Laboratories, Department of Anesthesiology, Department of Anesthesiology, University of Texas Medical Branch, Galveston, Texas.

**Keywords:** behavior, blast-induced neurotrauma, cerebral blood flow, cerebrovascular circulation, peroxynitrite, primary blast injury, reactive oxygen species, TBI

## Abstract

To determine the effects of mild blast-induced traumatic brain injury (bTBI), several groups of rats were subjected to blast injury or sham injury in a compressed air-driven shock tube. The effects of bTBI on relative cerebral perfusion (laser Doppler flowmetry [LDF]), and mean arterial blood pressure (MAP) cerebral vascular resistance were measured for 2 h post-bTBI. Dilator responses to reduced intravascular pressure were measured in isolated middle cerebral arterial (MCA) segments, *ex vivo*, 30 and 60 min post-bTBI. Neuronal injury was assessed (Fluoro-Jade C [FJC]) 24 and 48 h post-bTBI. Neurological outcomes (beam balance and walking tests) and working memory (Morris water maze [MWM]) were assessed 2 weeks post-bTBI. Because impact TBI (i.e., non-blast TBI) is often associated with reduced cerebral perfusion and impaired cerebrovascular function in part because of the generation of reactive oxygen and nitrogen species such as peroxynitrite (ONOO^−^), the effects of the administration of the ONOO^−^ scavenger, penicillamine methyl ester (PenME), on cerebral perfusion and cerebral vascular resistance were measured for 2 h post-bTBI. Mild bTBI resulted in reduced relative cerebral perfusion and MCA dilator responses to reduced intravascular pressure, increases in cerebral vascular resistance and in the numbers of FJC-positive cells in the brain, and significantly impaired working memory. PenME administration resulted in significant reductions in cerebral vascular resistance and a trend toward increased cerebral perfusion, suggesting that ONOO^−^ may contribute to blast-induced cerebral vascular dysfunction.

## Introduction

Traumatic brain injury (TBI) is one of the most common types of injuries among combatants in Operations Iraqi Freedom, Enduring Freedom, and New Dawn^1–5^ in part because of the high incidence of blast-induced TBI (bTBI). Although estimates of the prevalence of bTBI vary widely, some estimates are as high as 19–23%.^6–8^ As of January 2015, the Department of Defense reported that >73% of all United States military casualties were caused by explosive weaponry such as improvised explosive devices or mortars,^9,10^ making blast the most widespread cause of combat-related morbidity and mortality.^11–13^

Of the most widely accepted classifications of injury caused by blast, primary blast injury resulting from exposure to blast wave over/underpressures is the least well understood.^14^ Blast- and non-blast-induced TBI typically result in damage to both white and gray matter, with secondary cascades of cellular, molecular, and biochemical abnormalities, all of which may contribute to neuronal and/or glial injury.^3,15,16^

bTBI may also be associated with cerebral vascular injury^17^ similar to that resulting from impact (i.e., non-blast) TBI. TBI was followed by cerebral hypoperfusion in some patients^18–20^ and in experimental animals.^21–23^ Clinical TBI^24^ and fluid-percussion injury^25–27^ were also associated with impaired cerebral vascular responses to changes in arterial blood pressure (i.e., pressure autoregulation).^24–27^ Impact TBI also was associated with impaired cerebral vascular compensatory responses to changes in the partial pressures of carbon dioxide,^28–30^ oxygen,^31^ and hematocrit.^32^ Like impact TBI, bTBI resulted in some degree of cerebral vascular injury with recent evidence of level-dependent reductions in relative blood flow in the cortex and hippocampus of rats exposed to several shock-wave intensities in an air-driven shock tube.^33^ Both single and repeated blast overpressures resulted in impaired cerebral vascular endothelium-dependent dilation, a vascular pathology associated with extracellular matrix alterations and an increase in inflammatory cytokines for sustained periods post-blast.^34^ Additionally, blast exposure in animals caused blood–brain barrier breakdown,^35,36^ cerebral arterial vasospasm,^37^ increased vascular permeability,^38,39^ and decreased cerebral blood flow (CBF).^40^ The intensity of blast overpressures was also positively correlated with increased vascular leakage caused by disturbances in blood–brain barrier integrity,^40^ an increase in brain reactive oxygen species levels, astrocytosis, and cell apoptosis at several time points after blast exposure.^41^ Gama Sosa and colleagues^42^ reported a selective vascular pathology that was present 24 h after injury and persisted for months post-blast in brain regions with a seemingly undamaged neuropil. Ahmed and colleagues^43^ reported significant increases in plasma levels of vascular endothelial growth factor and von Willebrand factor, indicators of endothelial injury, that persisted for at least 42 days after repeated, mild bTBI.

Cerebral vascular dysfunction after impact TBI is caused, in part, by the effects of reactive oxygen/nitrogen species (e.g., superoxide anion, hydroxyl radicals, and peroxynitrite [ONOO^−^]),^44–50^ with previous studies indicating that ONOO^−^ exposure produced dose-dependent dilation in pial arteries in cats^47^ and constriction in isolated pressurized middle cerebral arteries (MCA) in rats.^51,52^

Several experimental studies have indicated that bTBI increased the production of nitric oxide^53,54^ and superoxide^55^ free radicals, suggesting that bTBI increases the formation of toxic ONOO^−^.^17^ ONOO^−^ is formed when excessive nitric oxide reacts with superoxide anion radicals.^56^ Cernak and colleagues^53^ reported that bTBI resulted in increases in the expression of inducible nitric oxide synthase messenger RNA and brain nitrite/nitrate levels, whereas Abdul-Muneer and colleagues^54^ reported that inducible nitric oxide synthase immunoreactivity increased within 1 h of mild (123 kPa) shock wave exposure. Cho and colleagues^55^ reported evidence of superoxide production (dihydroethidium fluorescence) starting 4 h and persisting for at least 2 weeks after 129.2 kPa ±3.0 (18.7 psi ±0.4) shock-wave exposure. Blast-induced increases in ONOO^−^ formation in the brain is further supported by evidence that bTBI was associated with increases in 3-nitrotyrosine immunoreactivity in the hippocampus and cortex.^57^ Abdul-Muneer and colleagues^54^ reported that mild bTBI was followed by increases in 3-nitrotyrosine immunoreactivity in rat brain microvessels whereas Hall and colleagues^48^ reported that the ONOO^−^ scavengers, penicillamine (Pen) and penicillamine methyl ester (PenME), improved outcome after TBI in mice. Together, these studies indicating that bTBI increases nitric oxide^53,54^ and superoxide^55^ levels and that 3-nitrotyrosine immunoreactivity in the brain and cerebral vasculature^54,57^ provides support for the hypothesis that ONOO^−^ formation is likely after bTBI, and contributes to blast-induced brain and cerebral vascular dysfunction.

We tested the hypothesis that bTBI, like non-blast TBI, results in cerebral hypoperfusion and impaired cerebral vascular reactivity by measuring relative cerebral perfusion *in vivo* (laser Doppler flowmetry [LDF]) and dilatory responses to reduced intravascular pressure in MCA segments obtained from rats subjected to mild bTBI using a compressed air driven shock tube. Additionally, we determined whether levels of bTBI sufficient to result in cerebral vascular dysfunction were also associated with cognitive dysfunction, neuronal injury, vestibulomotor deficits, working memory function, and increases in the numbers of Fluoro-Jade C (FJC)-positive cells in rats after bTBI. Lastly, we assessed the effects of ONOO^−^ scavenging on relative cerebral perfusion, mean arterial blood pressure (MAP), and cerebral vascular resistance in rats treated with PenME after bTBI.

## Methods

### Advanced Blast Simulator (ABS)

**FIG. 1.**
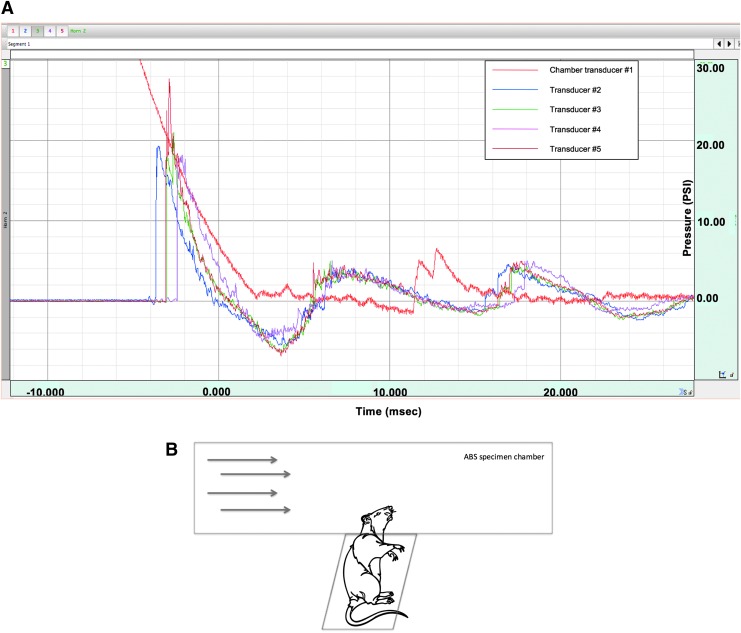
(**A**) Shock-wave over- and underpressures (in psi) produced by the Advanced Blast Simulator (ABS) versus time. Transducer 1, driver chamber; transducers 2 and 4, specimen chamber, up- and downwind from the rat head, respectively; transducer 3, specimen chamber directly over the rat head; transducer 5, specimen chamber adjacent to the rat head. (**B**) Direction and orientation of the experimental animal inside the ABS. When placed in the ABS, the animal was in a transverse prone position with the dorsal surface of the head perpendicular to the shock-wave direction (arrows). Color image is available online at www.liebertpub.com/neu

bTBI was produced by an ABS, a shock tube designed by David Ritzel (Dyn-FX Consulting, Ltd., Ontario, Canada) and produced by Steven Parks (ORA, Inc., Fredericksburg, VA). The ABS uses a compressed air driver to generate Freidlander-like^58^ over/underpressures (Fig. 1A). The driver chamber of the ABS was separated from the expansion section by Mylar^®^ membranes. Five piezoelectric pressure probe transducers (Piezotronics Inc., Buffalo, NY) located flush along the inside of the ABS were used to measure shock-wave pressures: one transducer in the driver chamber to measure the burst pressure and four in the specimen chamber with one right above the specimen tray, one 12 in. (30.5 cm) to the left of the tray, one 12 in. (30.5 cm) to the right of the tray, and one located on the specimen tray directly adjacent to the animal's head. Additional details about the ABS device parameters are available in Table 1.

**Table 1.** Preclinical Common Data Elements for ABS bTBI

*Animal characteristics*	
Species	*Rattus norvegicus*
Age range	3–5 mo. prior to TBI
Sex	Male
Animal vendor	Charles River
Strain	Sprague-Dawley
Weight range	350–480 g pre-TBI
*Animal history*	
Pre-TBI housing	Group housed; 12 h light/dark cycle; food and water *ad libitum*; AAALAC accredited animal care facility maintained to USDA standards
Anesthetic type	Isoflurane (4% for induction, 2% for maintenance), intubated and mechanically ventilated
Anesthetic route	Inhaled
Analgesia type	Acetaminophen suppository
Injury severity	Mild (blast)
Number of injury exposures	Single
Post-TBI housing	Group housed unless separated for fighting; 12 h light/dark cycle; food and water *ad libitum*; AAALAC accredited animal care facility maintained to USDA standards
Euthanasia type	4% Isoflurane followed by decapitation
*Assessments and outcomes*	
Acute neurological assessment	Combined neuroscore^156^
Righting reflex response time	Mild = ≤ 7 min; moderate = 8–14 min; severe = > 14 min
Learning and memory tests	Morris water maze
Sensory/motor tests	Beam walk; beam balance
Anxiety and depression tests	N/A
Histopathology	Cellular/neuronal Injury
*Injury model characteristics*	
Injury model	Advanced Blast Simulator (ABS) TBI
Device manufacturer	ORA, Inc., Fredericksburg, VA
Animal stabilization method	Rat's dorsal scalp is perpendicular to the ABS blast wave with only the cranium located inside the ABS device and supported by a sling to reduce head movement. The rest of the body lies in a left lateral position outside the ABS device on a specimen tray that locks into it the blast chamber.
Impact location side	Central dorsal
*ABS blast TBI*	
Blast-induced delivery device	ABS device is a shock tube designed by David Ritzel (Dyn-FX Consulting, Ltd. Ontario, Canada) and produced by Steve Parks (ORA, Inc., Fredericksburg, VA)
Pressure wave type	Single pulse blast waves (Friedlander-style over- and underpressure waves)
Detonation type	Shockwaves produced using compressed gas driver and Mylar® membranes
Detonation material quantity	Mylar sheets (4)
Driver gas	Compressed air
Pressure wave medium	Air
Distance from detonation	6 feet, 7 inches (2 m)
Blast tube or column area	90 square inches (581 cm^2^)
Blast tube length	14 feet (4.27 m)
Shock tube driven section length	10 inches (254 mm)
Membrane thickness	0.016 inches (0.4 mm/sheet)
Membrane burst method	Non-debris complete rupture
Membrane burst pressure	179.33 psi ±3.0 (1236.4 kPA ±20.7)
Tube end configuration	Reflected wave suppressor
Distance between animal and tube	Rat's head is inside of tube
Animal orientation to blast wave	Perpendicular
Overpressure peak	20.88 psi (138 kPa)
Overpressure rise time	0.37 ms ±0.006
Overpressure wave duration	3.50 ms ±0.063
Impulse	230.34 Pa/sec ±8.9
Pressure sensor type	Piezoelectric pressure probe transducers
Pressure sensor sampling frequency	20 μs time sample ≥50 kHz sample rate
Incident Pressure Time History	<2 μs
Body exposure	Head only
Protective shielding location	N/A
Protective shielding type	N/A
Primary blast effects	Absence of external injury: occluded blood vessels, cerebral vasospasm, subarachnoid hemorrhage, tympanic membrane rupture, neuronal death/degeneration
Secondary blast effects	N/A
Tertiary blast effects	N/A
Quaternary blast effects	N/A
Systemic injuries	None
Extracranial injuries	None
Pre-bTBI surgical procedures	Isoflurane (4% initial, 2% to maintain until blast injury), intubated, mechanically ventilated, and the top of the scalp is shaved
Post-bTBI surgical procedures	Measurement of duration of righting reflex suppression and removal of intubation tube

AAALAC, *Association for Assessment and Accreditation of Laboratory Animal Care;* USDA, United States Department of Agriculture; TBI, traumatic brain injury; bTBI, blast-induced TBI.

### ABS injury and animal preparation

All experimental protocols were approved by the Institutional Animal Care and Use Committee of the University of Texas Medical Branch, an Association for Assessment and Accreditation of Laboratory Animal Care accredited facility. All animals were housed under controlled environmental conditions and allowed food and water *ad libitum*. Adult, male, Sprague–Dawley rats (Charles River Laboratories, Wilmington, MA) ranging in weight from 350 to 480 g were anesthetized (4% isoflurane), intubated and ventilated (2.0% isoflurane) in O_2_/room air (80:20) using a volume ventilator (Small Animal Ventilator, Harvard Apparatus, Inc., Holliston, MA). Core body temperature was monitored using a rectal telethermometer (Thermalert Monitoring Thermometer, Physitemp Instruments, Inc., Clifton, NJ) and maintained within normal limits using a thermostatically controlled water blanket (Mul-T-Pad Temperature Therapy Pad, Gaymar Industries, Inc., Orchard Park, NY). After intubation, the scalp was shaved, foam plugs were placed in each ear, and the animal was secured on the specimen tray with Velcro^®^ straps in a transverse prone position with the head supported at right angles to the direction of the shockwave by a leather sling suspended between two supports. When the specimen tray was placed in the ABS, only the rat's head was exposed to the shockwave (Fig. 1B). After the rat was secured to the specimen tray, the isoflurane was temporarily discontinued, the ventilator hoses were detached but the rat remained intubated, the specimen tray was locked into the ABS and, at the return of a withdrawal reflex to paw pinch, the rat was subjected to bTBI (19.6 psi ±1.8, 135 kPa ±12.4) or sham bTBI. Shock-wave overpressures had a mean rise time of 0.37 ms ±0.006 and a duration of 3.50 ms ±0.063, calculated using the slope and y-intercept formula. After injury, the animal was removed from the ABS, the duration of suppression of the righting reflex was recorded, the animal was reconnected to the ventilator, and anesthesia with isoflurane was resumed. For all sham animals, the preparatory procedure previously stated was followed, but the rat was not subjected to bTBI. Rats were intubated and mechanically ventilated for the measurements of relative cerebral perfusion to maintain constant anesthetic levels. For the sake of consistency and to make the separate experiments as similar as possible, all rats were intubated and ventilated.

### Measurements of MCA diameters

Thirty or sixty mintes after ABS bTBI (*n* = 6/group) or sham (*n* = 6/group) injury, the isoflurane level was increased to 4% for a minimum of 5 min, the rat was decapitated, the brain was removed, and MCA segments were collected and mounted in an arteriograph (Living Systems Instrumentation, Inc., Burlington, VT)^52,59^ within 15–20 min of harvest by an investigator blinded to the experimental group. The arterial segment was bathed in physiologic salt solution composed of 130 mM NaCl, 4.7 mM KCl, 7 mM MgSO_4_, 1.17 mM H_2_O, 5 mM glucose, 1.5 mM CaCl_2,_ and 15 mM NaHCO_3_ maintained at 37°C and equilibrated for 60 min with intravascular pressure set at 50 mm Hg. The MCA segments were viewed using an inverted microscope equipped with a video camera and video scaler. Dilator responses were confirmed by decreasing intravascular pressure in 20 mmHg increments with a 10 min equilibration period at each level before the diameter was measured.

### Measurements of relative cerebral perfusion using LDF

Rats were anesthetized, intubated, and ventilated as described, and a tail artery was cannulated with polyethylene (10) tubing. The animal was placed in a stereotaxic frame and the scalp shaved. The midline scalp was incised, reflected and a .25 in. (0.6 cm) portion of the skull lateral to the midline over the frontal-parietal cortex thinned using an air-cooled dental drill. A fiberoptic needle probe was positioned over the thinned area away from large blood vessels, and shielded from external light. Baseline cerebral perfusion and MAP were measured, the LDF probe was removed, the edges of the scalp were sutured, foam plugs were placed into each ear, and the animal was removed from the stereotaxic frame and secured on the ABS specimen tray. Anesthesia was temporarily discontinued and, immediately after the return of a withdrawal reflex to paw pinch, the rat was subjected to ABS bTBI (*n* = 12) or sham (*n* = 10) injury as described. Immediately after injury, the animal was removed from the ABS and reconnected to the ventilator, anesthesia was resumed (2.0% isoflurane), the animal was re-secured to the stereotaxic frame, the were sutures cut, the scalp was reflected, and the LDF needle probe was repositioned over the same thinned skull area. A temperature probe was placed deep into the temporalis muscle. Cerebral perfusion and arterial blood pressure recordings were continued for 2 h post-injury. Relative cerebral perfusion was calculated and expressed as a percent of pre-bTBI baseline. Cerebral vascular resistance was calculated from MAP and cerebral perfusion (cerebral vascular resistance = MAP/LDF).

### Technical considerations-LDF

Laser Doppler perfusion measurements are expressed as a percentage of baseline values. Ideally, the LDF probe remains in the same location for the duration of the measurements. However, in our studies, baseline LDF measurements were recorded and the rats were removed from the stereotaxic frame, subjected to ABS bTBI or sham injury, and then returned to the stereotaxic frame. Although we attempted to replace the probe in exactly the same location from which the baseline measurements were made, we may have been unable to do so in some cases. In order to compensate for the possibility of misplaced probes in both the sham and bTBI groups, we excluded all animals in which the first measurement after the probe was replaced (5 min post-blast time point), which yielded LDF values 20% higher or lower than baseline in the absence of comparable changes in MAP (five sham rats and three bTBI rats were excluded). However, it is important to note that relative cerebral perfusion was significantly (*p* < 0.02, bTBI vs. sham) reduced by mild bTBI even if all animals were included in the calculations of relative cerebral perfusion. Additionally, it is possible that thinning of the skull for LDF measurements may have compromised skull integrity, thus contributing to injury induction/severity. However, the scalp covering the thinned area was tightly sutured before administration of blast injury. We observed no evidence of skull fracture in the rats subjected to bTBI. Although some minor surface blood was observed under the thinned skull in 4 out of the 12 rats subjected to bTBI, no hemorrhage was observed in 8 of the 12.

### Histopathology

Rats were subjected to ABS bTBI (*n* = 6/group) or sham (*n* = 6/group) injury, the duration of suppression of the righting reflex was measured, and 24 or 48 h later, the rats were anesthetized with 4.0% isoflurane for a minimum of 5 min and decapitated. The brains were removed and immediately frozen on dry ice and stored at -80°C. The brains were sectioned (10 μm) on a cryostat (Leica CM1860, Leica Biosystems, Inc., Buffalo Grove, IL) and every 25th section was mounted on microscope slides (Fisherbrand™ Superfrost™ Plus slides, Fisher Scientific Co., Pittsburgh, PA). Sections were immersed in 75% ethanol for 1 min, in Milli-Q^®^ H_2_O for 1 min. and in cresyl violet for 15–20 sec at room temperature. Sections then were washed in Milli-Q H_2_O twice for 30 sec each then immersed in FJC (0.0001% in Milli-Q H_2_O with 0.1% acetic acid vehicle) for 4 min. Sections were removed from the FJC and washed in Milli-Q H_2_O three separate times for 1 min each, in 95% ethanol for 30 sec, and then in 100% ethanol for 30 sec. Lastly, sections were immersed in xylene twice for 3 min each, then allowed to air dry overnight in a darkened fume hood. Two investigators, who were blinded to the experimental groups, counted 30 slides with two sections mounted on each slide (an average of 60 brain sections for each animal). Ten sections (five slides) were taken from the region corresponding to the frontal lobe, 40 sections (20 slides) were taken from the parietal/temporal lobe region/s, and 10 sections (five slides) were taken from the occipital lobe/cerebellum region. FJC-positive cells in each section were viewed using an imaging system monitor connected to an Olympus BX51 research system microscope (Olympus Corporation, Tokyo, Japan) using a filter system suitable for visualizing fluorescein or fluorescein isothiocyanate. FJC-positive cells were summed across all sections for each individual region. The mean of the two investigators' counts was calculated to get a total mean count for each whole brain. Some sections were stained with hematoxylin and eosin (H&E) to determine whether mild bTBI resulted in histological evidence of injury (e.g. intraparenchymal hemorrhage, ventricular enlargement).

### Technical considerations-histopathology

Although FJ is widely used to stain injured and/or dying neuronal cells,^60–64^ there is evidence that FJ-positive cells may be injured but not necessarily dying,^65^ and FJ may stain non-neural cells (e.g., activated microglia, astrocytes) under circumstances in which it is combined with specific markers for detection of glial fibrillary acidic protein or activated CD68 microglia.^66^

### Assessment of behavioral and cognitive function

Each animal was randomly assigned to receive ABS bTBI (*n* = 10) or sham (*n* = 10) injury, trained on beam walk^67–69^ and beam balance^67,68,70^ tasks the day before bTBI or sham injury and on the day of blasting prior to injury, subsequently tested on the beam walk and beam balance tasks on post-injury days 1–5, and then tested on the Morris water maze (MWM) task on post-injury days 11–15. The beam walk task involved training rats to traverse an ∼3 ft. (1 m) long, 1 in. (2.5 cm) wide solid pine beam with a darkened goal box attached at the far end and a lamp and white noise generator as an aversive stimulus at the starting end. Dulled nails (1.75 in. [4.4 cm] length) acting as distracting obstacle pegs were alternately staggered near the edges along the beam to provide some negotiation difficulty. The time required for the animal to reach the goal box from the starting point was recorded for three successive trials. For the beam balance task, rats are trained to balance on a plywood beam ∼1 ft. (0.3 m) in length and .5 in. (1.3 cm) wide that is open ended, with a whiteboard barrier on the other end. Each animal was scored on its ability to balance on the beam for 60 sec on three separate trials. The MWM task^71–74^ utilized a white, circular pool 6 ft. (2 m) in diameter filled to a depth of ∼2.5 ft.) (0.8 m), which contained a transparent goal platform (4.5 in. [11.4 cm] diameter) located just below the water's surface. External cues consisting of arrows and rectangles are attached to each of the four walls in the testing room for spatial reference. Each animal received four pairs of timed trials per day, for 5 consecutive days. For each pair of trials, the entry point and platform locations were randomized, while visual cues located on the walls of the testing chamber remained constant throughout each day. For each trial, the animal was placed in the maze facing the pool wall and given 120 sec to locate and climb onto the hidden platform. If the animal was not able to locate the platform by the allotted time at the end of the first trial, it was placed on the platform for 30 sec before being returned to the start position for the second trial. Between the pairs of timed trials, the animals were kept in a heated warming box. During the 4 min interval between pairs of trials, both the start position and the goal location were changed. Movement within the maze was recorded with a video camera, a video scanning unit, and the SMART tracking computer software (San Diego Instruments, Inc., San Diego, CA).

### Effects of ONOO^−^ scavenger administration on relative cerebral perfusion using LDF

Rats were prepared for measurements of relative cerebral perfusion and MAP as described. Anesnthesia was discontinued following baseline LDF and MAP measurements and, immediately after the return of a withdrawal reflex to paw pinch, the rats were subjected to ABS bTBI (*n* = 8), ABS bTBI + PenME treatment (*n* = 8), or sham (*n* = 8) injury. Immediately after injury, anesthesia (2.0% isoflurane) was resumed, the rat was returned to the stereotaxic frame, the scalp reflected, and the LDF needle probe was repositioned over the thinned skull. A temporal temperature probe was then placed deep into the temporalis muscle. Five minutes post-bTBI, 10 mg/kg of PenME was administered in 0.1 mL saline vehicle through the cannulated tail vein. Measurements of cerebral perfusion and arterial blood pressure were continued for 2 h post-bTBI. Relative cerebral perfusion was calculated and expressed as a percent of pre-bTBI baseline. Cerebral vascular resistance was calculated from MAP and related cerebral perfusion (cerebral vascular resistance = MAP/LDF).

### Technical considerations-ONOO^−^ scavenger administration

Pen is a stoichiometric scavenger that, through a series of proton transfer reactions, inactivates ONOO^−^ by converting it to the reactive nitrogen species peroxynitrous acid and then to nitro-penicillamine and water.^48^ Using a radioimmunoassay that measured the protection of cyclic adenosine monophosphate (cAMP) from ONOO^−^, Althaus and colleagues^75^ reported that Pen was the most effective of the 10 ONOO^−^ scavengers that they tested (e.g., cystoamino, cysteine, cysteine methyl ester, cystoamino methyl ester, Pen). Whereas Pen has limited blood– brain barrier permeability and acts intravascularly, PenME is a lipophilic, blood–brain barrier permeable ONOO^−^ scavenger that can act extravascularly.^48^ The dose of PenME was selected based on a previous report that 10 mg/kg produced dose-related improvements in early neurological recovery.^48^

### Statistical analysis

Statistical analysis was performed using GraphPad Prism 5 software, (GraphPad software version 5.00, San Diego, CA). The response to changes in intravascular pressure was assessed by calculating percent change from baseline (100 mm Hg) for each level of intra-arterial pressure (80, 60, 40, and 20 mm Hg). Unpaired Student's *t* tests were used to evaluate differences between the bTBI and sham group baselines. Differences in MCA dilator responses between bTBI and sham groups were assessed using a repeated one way analysis of variance (ANOVA) Dunnett's multiple comparisons test and a Bartlett's test for equal variance. Relative cerebral perfusion, MAP, and cerebral vascular resistance data in both the untreated and PenME-treated studies were analyzed using unpaired Student's *t* tests and two-way ANOVA after calculating percent change from baseline for each respective parameter. For the whole brain analysis, the numbers of FJC-positive cells counted by two investigators blinded to group (i.e., sham vs. bTBI) were averaged for each of the four groups (24 and 48 h sham, 24 and 48 h bTBI). Means for the whole brain cells counts between the 24 and 48 h sham and bTBI groups were compared using one way ANOVAs. For the regional analysis, the numbers of FJC-positive cells counted by the two investigators were averaged for each region (frontal, parietal/temporal, and occipital) in each of the four groups. The numbers of FJC-positive cells in the frontal, parietal/temporal, and occipital regions 24 and 48 hr post-bTBI were compared using two way ANOVAs followed by post-hoc testing used to identify significant differences among injury groups and regions. A repeated measure two way ANOVA was performed on the differences in the MWM latencies to the goal platform between the first and second trials of each successive day between the two groups as a whole. Because beam balance scores were ordinal, those data were analyzed using the Mann–Whitney test.

Because of the reduction in statistical power that results from repeated testing, comparisons at each specific pressure time point in the MCA experiments (e.g., between 100 and 80 or between 60 and 40) or between individual days between the two groups in the beam walk, beam balance, and MWM trials (e.g., between days 1 and 5 or days 12 and 14) were not conducted. Significance was accepted at the *p* ≤ 0.05 level. All data in the text, table, and figures are expressed as means ± standard errors of the means.

## Results

### ABS bTBI

The mean ABS bTBI overpressures for all experiments was 19.6 psi ±1.8 (135 kPa ±12.4).

### Righting reflex

**FIG. 2.**
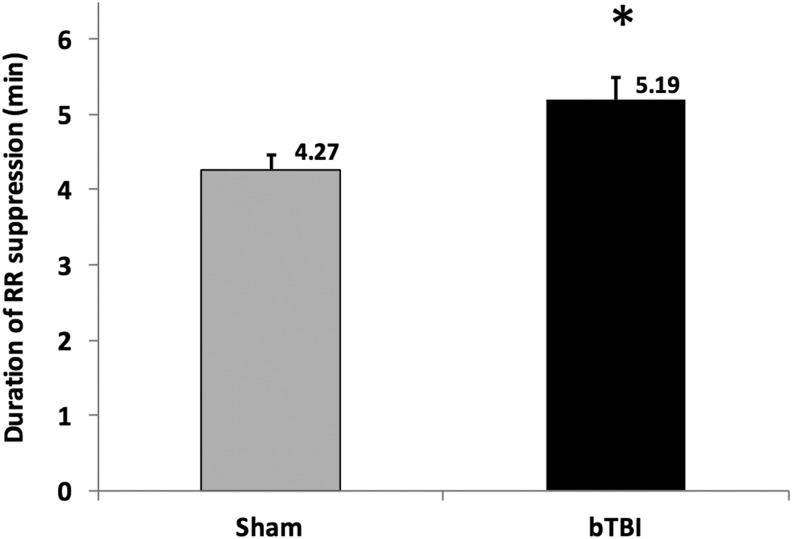
Effects of blast-induced traumatic brain injury (bTBI) on righting reflex (RR) suppression (*n* = 46/group). Mean duration of RR suppression for the bTBI group (5.19 min ±2.1) was significantly longer than in the sham group (4.27 min ±1.6). This duration of RR suppression is considered within the range of mild bTBI injury. Values are means ± SEM. *p = 0.007 versus sham.

The mean duration of righting reflex suppression for all of the rats subjected to ABS bTBI shock-wave exposure (5.19 min ±2.1) was significantly higher (*p* = 0.007, bTBI vs. sham) than for the sham group (4.27 min ±1.6) (Fig. 2).

### Effects of ABS bTBI on dilator responses to reduced intravascular pressure in isolated MCA segments

**FIG. 3.**
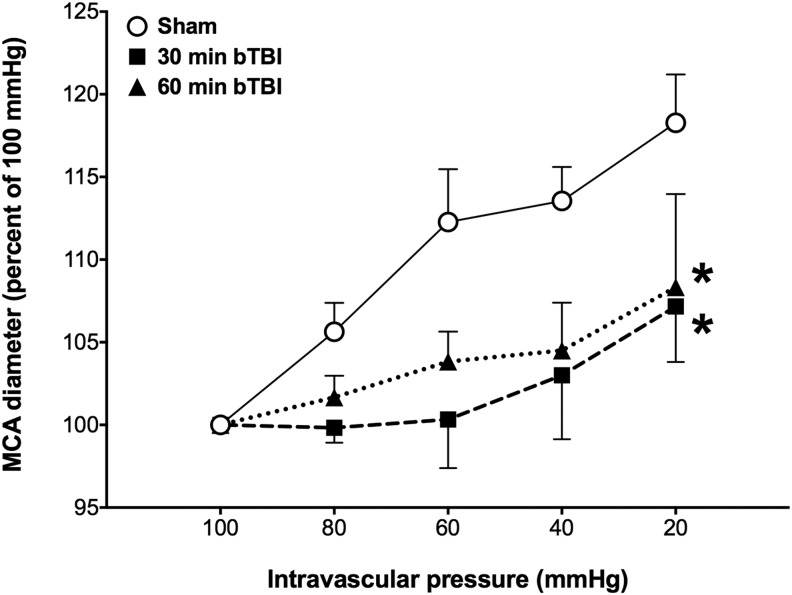
Effects of blast-induced traumatic brain injury (bTBI) on middle cerebral arterial (MCA) responses to reduced intravascular pressure (*n* = 6/group). Dilator responses to progressive reductions in intravascular pressure were significantly reduced in the 30 min and 60 min bTBI groups. Values are plotted as means ± SEM. *p < 0.05 versus sham.

In both the 30 and 60 min sham groups, MCA diameters increased above baseline as intraluminal pressure was reduced from 100 to 20 mm Hg. Dilator responses to progressive reductions in intravascular pressure were significantly reduced in the 30 min (*p* = 0.01, bTBI vs. sham) and 60 min (*p* = 0.02, bTBI vs. sham) ABS bTBI groups after blast exposure (Fig. 3).

### Effects of ABS bTBI on MAP, relative cerebral perfusion, and cerebral vascular resistance

**FIG. 4.**
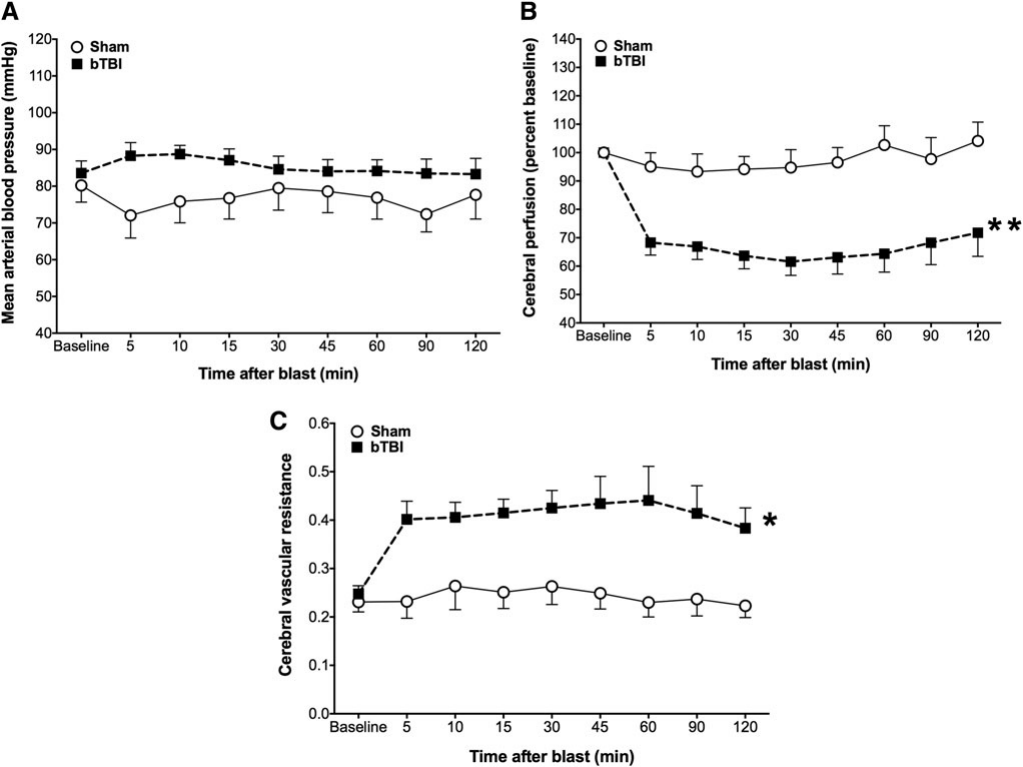
Effects of blast-induced traumatic brain injury (bTBI) (*n* = 12) or sham bTBI (*n* = 10) on mean arterial blood pressure (MAP), relative cerebral perfusion, and cerebral vascular resistance (CVR). (**A**) Although there was a trend toward elevated MAP in the bTBI group for at least 2 h after injury, the difference in MAP between the bTBI and sham group was not significant (*p* = 0.11, bTBI vs. sham). (**B**) Relative cerebral perfusion was significantly reduced in the bTBI group compared with the sham group for at least 2 h after bTBI whereas (**C**) CVR was significantly elevated in the bTBI group compared with the sham group for at least 2 h after bTBI. Values are plotted as means ± SEM. *p < 0.001 versus sham; **p < 0.0001 versus sham.

Although there was a trend toward elevated MAP in the ABS bTBI group, the differences were not statistically significant (*p* = 0.11, bTBI vs. sham) (Fig. 4A). In contrast, cerebral perfusion was significantly reduced (*p* < 0.0001, bTBI vs. sham) (Fig. 4B) and cerebral vascular resistance was significantly elevated (*p* = 0.00042, bTBI vs. sham) (Fig. 4C) for at least 2 h post-bTBI.

### Effects of ABS bTBI on cell injury in the brain

**FIG. 5.**
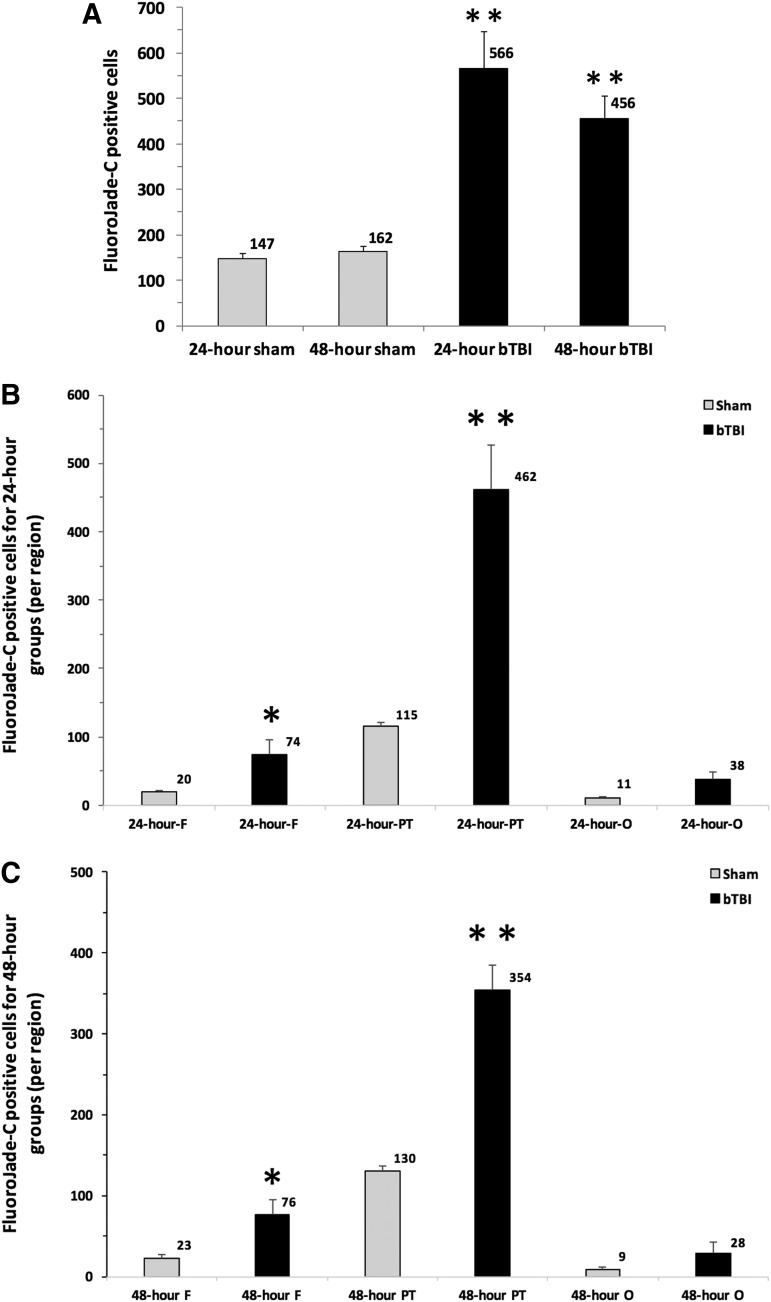
Effects of blast-induced traumatic brain injury (bTBI) on cell injury in the brain (*n* = 6/group). (**A**) The number of Fluoro-Jade C (FJC) positive cells counted throughout 60 sections taken from the whole brain was significantly greater 24 and 48 h after bTBI compared with both the 24 and 48 h sham groups. (**B**) The numbers of FJC positive cells in the frontal (F) and parietal/temporal lobe (PT) region were significantly greater than in the sham group's F region and PT region. However, the bTBI group's occipital (O) region was not significantly different from the sham group's O region (*p* = 0.057, bTBI O vs. sham O). (**C**) The numbers of FJC positive cells in the frontal (F) and parietal/temporal lobe (PT) region were significantly greater 48 h post-bTBI in the bTBI group than in the sham group, but were not significantly different from sham in the occipital (O) region (*p* = 0.320, bTBI O vs. sham O). Values are plotted as means ± SEM. *p < 0.05 versus sham and F; **p < 0.001 versus sham.

The total numbers of FJC-positive cells were significantly greater 24 h (*p* = 0.0004, 24 h bTBI vs. 24 h sham) and 48 h (*p* = 0.0001, 48 h bTBI vs. 48 h sham) after ABS bTBI injury compared with sham injury (Table 2, Fig. 5A). The mean number of FJC-positive cells in the frontal and parietal/temporal regions were significantly greater 24 h (Fig. 5B) and 48 h (Fig. 5C) post-injury in the ABS bTBI group than in the sham group.

**Table 2.** Effects of ABS bTBI on the Numbers of Fluoro-Jade-Positive Cells in Frontal, Parietal/Temporal, and Occipital Brain Regions

*Brain region*	*Time*	*bTBI*	*Sham*	p *value (bTBI vs. sham)*
Frontal	24 h	74 ± 21	20 ± 2.3	0.037
	48 h	76 ± 19.5	23 ± 4.8	0.018
Parietal/temporal	24 h	462 ± 65.6	115 ± 7	0.003
	48 h	354 ± 31.3	130 ± 7.1	0.0005
Occipital	24 h	38 ± 10.6	11 ± 2.4	0.057
	48 h	28 ± 15.1	9 ± 2.4	0.320
Total	24 h	566 ± 20.9	147 ± 10.9	0.0004
	48 h	456 ± 17.9	162 ± 11.4	0.0001

bTBI, blast-induced traumatic brain injury.

### Effects of ABS bTBI on vestibulomotor and cognitive function

**FIG. 6.**
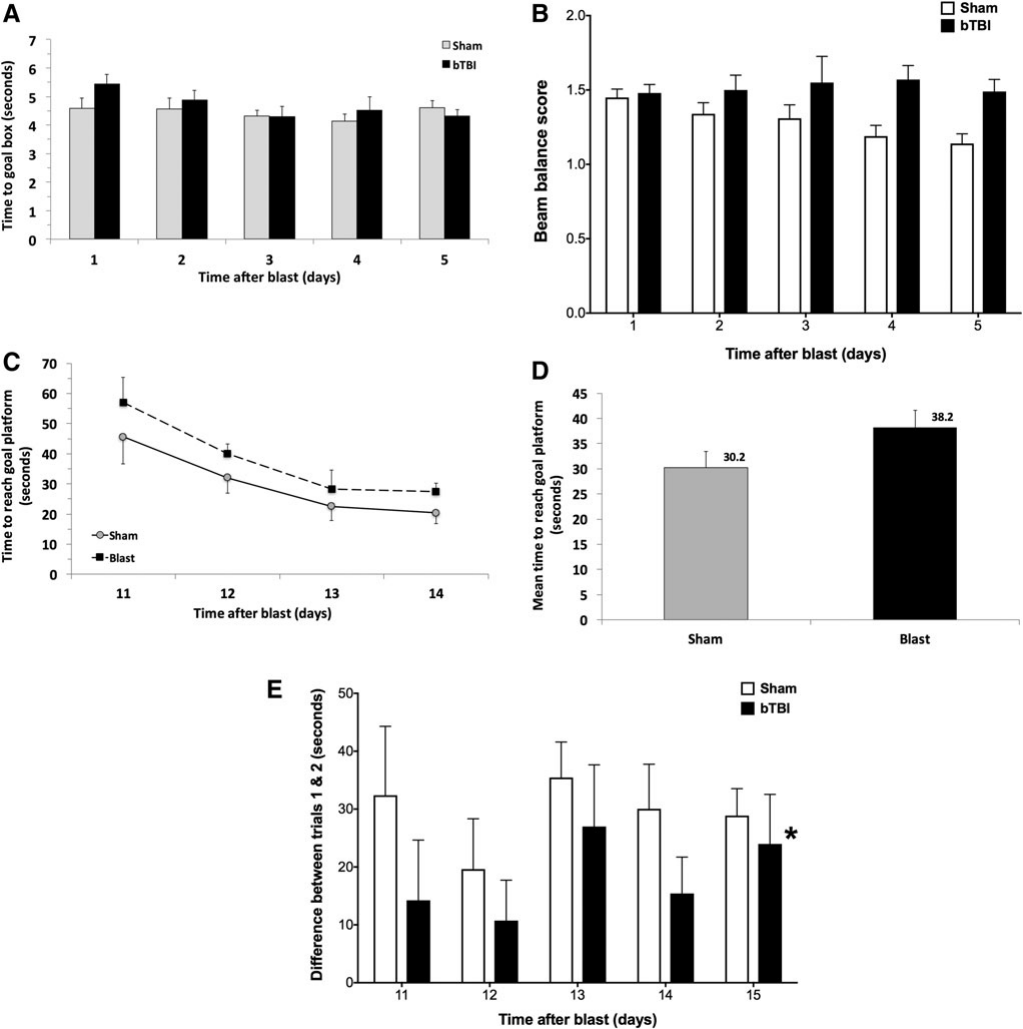
Effects of blast-induced traumatic brain injury (bTBI) on beam walk, beam balance, and working memory performance (*n* = 10/group). (**A**) Beam walk performance did not differ significantly between the bTBI and sham groups (*p* = 0.2, bTBI vs. sham). (**B**) Beam balance performance did not differ significantly between the bTBI and sham groups (*p* = 0.06, bTBI vs. Sham). (**C**) bTBI and sham group times to locate the goal platform on post-injury days 11–14. (**D**) bTBI and sham group latencies combined across all post-injury days. Neither the individual day nor the combined latencies differed significantly between the bTBI and sham groups (*p* = 0.067, bTBI vs. sham). (**E**) Differences in latencies to the goal platform between the first and second trials for each pair of trials in the bTBI and sham groups (*n* = 10/group). One way analysis of variance (ANOVA) indicated that the differences in latencies combined across all 5 days were significantly longer in the sham group than in the bTBI group, suggesting that bTBI resulted in significantly impaired working memory. Values are plotted as means ± SEM. *p < 0.01 versus sham.

Beam walk performance was not significantly different between the ABS bTBI and sham groups (*p* = 0.2, bTBI vs. sham) (Fig. 6A) even though there appeared to be a trend of improved performance between days 1 and 4 in the sham group. Although there was a trend toward impaired beam balance performance in the bTBI group and improved performance in the sham group throughout all days tested, the differences were not significantly different (*p* = 0.06, bTBI vs. sham) (Fig. 6B). Similarly, although there was a trend toward longer MWM latencies to the goal platform in the bTBI group (38.2 sec ±3.4) compared with the sham group (30.2 sec ±3.2) across days 11 through 14, these latencies were not significantly different between the sham and bTBI groups (*p* = 0.067, sham vs. bTBI) (Fig. 6C and D, Table 3). Swim speed values also were not significantly different between the bTBI (0.239 m/sec ±0.003) and sham groups (0.219 m/sec ±0.004) (*p* = 0.32, bTBI vs. sham). The intertrial (trial 1 vs. trial 2 for each pair of trials) differences in latencies were significantly longer for the sham group than for the bTBI group on each post-injury day tested (*p* = 0.01, bTBI vs. sham) (Fig. 6E), indicating that bTBI resulted in impaired working memory.

**Table 3.** Effects of ABS bTBI on Vestibulomotor and Cognitive Functions

	*Swim speed*	*MWM latencies*	p *value*
Beam walk (bTBI vs. sham)			0.2
Beam balance (bTBI vs. sham)			0.06
Days 11–14 after bTBI (bTBI vs. sham)	0.239 m/sec ±0.003		0.32
Days 11–14 after Sham	0.219 m/sec ±0.004		
Days 11–14 after bTBI (bTBI vs. sham)		38.2 sec ±3.4	0.067
Days 11–14 after sham		30.2 sec ±3.2	
Inter-trial latency differences (bTBI vs. sham)			0.01

ABS, Advanced Blast Simulator; bTBI, blast-induced traumatic brain injury; MWM, Morris water maze.

### Effects of ABS bTBI on MAP, relative cerebral perfusion, and cerebral vascular resistance after PenME administration

**FIG. 7.**
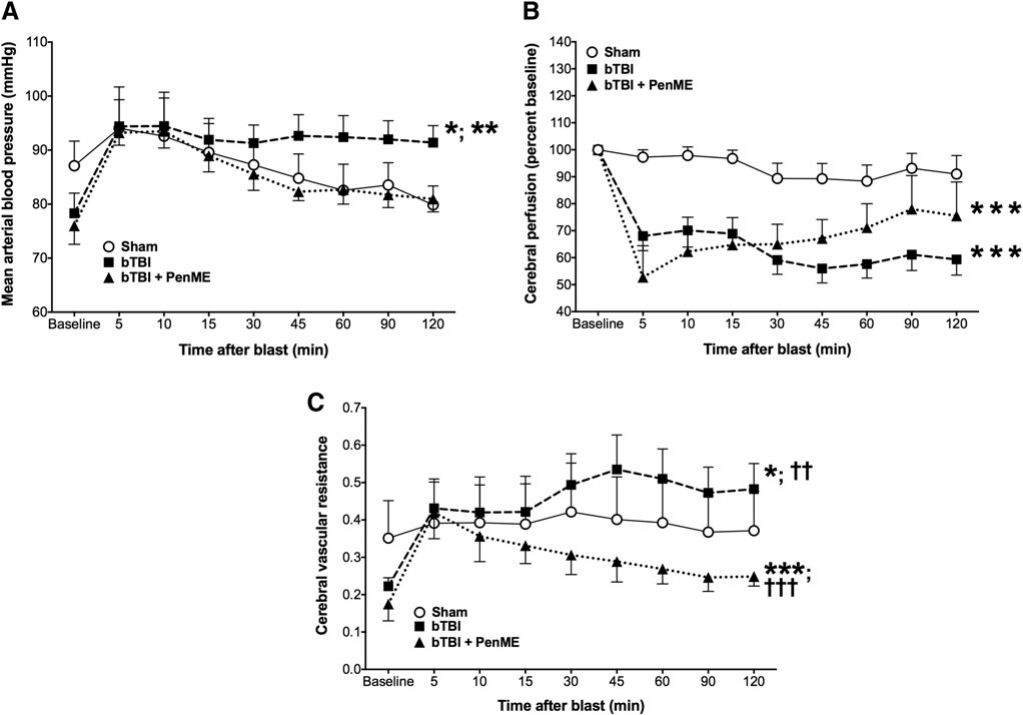
Effects of blast-induced traumatic brain injury (bTBI) on mean arterial blood pressure (MAP), relative cerebral perfusion, and cerebral vascular resistance (CVR) after penicillamine methyl ester (PenME) treatment (*n* = 8/group). (**A**) MAP was significantly elevated in the bTBI group compared with the sham and bTBI+PenME groups. However, there were no statistically significant differences between the sham and bTBI+PenME groups (*p* = 0.24, sham vs. bTBI+PenME). (**B**) Relative cerebral perfusion was significantly reduced in both the bTBI and bTBI+PenME groups compared with sham but did not differ significantly between the bTBI and bTBI+PenME groups (*p* = 0.11, bTBI vs. bTBI+PenME). (**C**) CVR was significantly elevated in the bTBI group compared with the sham and bTBI+PenME groups, but was significantly reduced in the bTBI+PenME group compared with both the sham and bTBI groups. Values are means ± SEM. *p < 0.01 versus sham; **p < 0.001 versus bTBI+PenME; ***p < 0.0001 versus sham; ^**††**^*p* < 0.0001 versus bTBI+PenMe; ^**†††**^*p* < 0.0001 versus bTBI.

MAP was significantly elevated in the bTBI group compared with the sham group (*p* < 0.01, bTBI vs. sham) and the bTBI + PenME group (*p* = 0.001, bTBI vs. bTBI + PenME) (Table 4). However, there were no significant differences between the sham and bTBI + PenME groups (*p* = 0.24, sham vs bTBI + PenME) (Fig. 7A), suggesting that PenME reduced the blast-related increases in MAP. Relative cerebral perfusion was significantly reduced in the bTBI (*p* < 0.0001, bTBI vs. sham) and bTBI + PenME groups (*p* < 0.0001, bTBI + PenME vs. sham) for at least 2 h after mild bTBI. Although there was a trend toward increased perfusion in the bTBI + PenME group beginning 30 min post-injury, there were no statistically significant differences in perfusion between the bTBI and bTBI + PenME groups (*p* = 0.11, bTBI vs. bTBI + PenME) (Fig. 7B). Cerebral vascular resistance was significantly elevated in the bTBI group compared with the sham (*p* < 0.01, bTBI vs. sham) and bTBI + PenME groups (*p* < 0.0001, bTBI vs. bTBI + PenME). Interestingly, cerebral vascular resistance was significantly lower in the bTBI + PenME group than in the sham group (*p* < 0.0001, bTBI + PenME vs. sham) and the bTBI group (*p* < 0.0001, bTBI + PenME vs. bTBI) for at least 2 h post-bTBI (Fig. 7C).

**Table 4.** Effects of ABS bTBI on MAP, Relative Cerebral Perfusion and CVR after PenME Administration

	p *value*
MAP (bTBI vs. sham)	<0.01
MAP (bTBI vs. bTBI+PenME)	0.001
MAP (sham vs. bTBI+PenME)	0.24
Relative cerebral perfusion (bTBI vs. sham)	<0.0001
Relative cerebral perfusion (bTBI vs. bTBI+PenME)	0.11
Relative cerebral perfusion (sham vs. bTBI+PenME)	<0.0001
CVR (bTBI vs. sham)	<0.01
CVR (bTBI vs. bTBI+PenME)	<0.0001
CVR (sham vs. bTBI+PenME)	<0.0001

ABS, Advanced Blast Simulator; bTBI, blast-induced traumatic brain injury; MAP, mean arterial blood pressure; CVR, cerebral vascular resistance; PenME, penicillamine methyl ester

## Discussion

Our results indicated that mild bTBI was associated with significant reductions in relative cerebral perfusion and increases in cerebral vascular resistance that occurred within 5 min and persisted for at least 2 h post-bTBI. Relative cerebral perfusion was reduced by ∼40%, a perfusion level unlikely to result in permanent neuronal injury.^76^ However, Mies and colleagues^77^ reported that CBF values <80 mL/min/100 g may be associated with selective neuronal loss in rats. Further, inhibition of protein synthesis began at cerebral levels <80 mL/min/100 g^78^ and, at 55 mL/min/100 g, protein synthesis was reduced by 50%.^79^ Glutamate release occurred in rats when CBF fell below 48% of normal values.^80^ Therefore, although mild bTBI did not reduce cerebral perfusion to levels typically associated with ischemic neuronal pannecrosis, blast-induced hypoperfusion may have been sufficient to contribute to the type of selective neuronal injury that we observed. Additionally, reduced perfusion may have impaired neuronal functioning by reducing protein synthesis and/or increasing the release of excitatory neurotransmitters. Although it is possible that neuronal injury or dysfunction caused by blast-related cerebral hypoperfusion contributed to working memory dysfunction, because our measurements of relative cerebral perfusion and working memory function were performed in separate animals at different time intervals, support for this hypothesis would require further experiments specifically designed to address the question.

These studies also revealed that mild bTBI significantly increased cerebral vascular resistance, an observation consistent with previous reports of blast-induced vasospasm.^81,82^ Mild bTBI also resulted in significant reductions in dilator responses to reduced intravascular pressure that persisted for at least 1 h post-blast, suggesting that mild bTBI results in significant impairment of cerebral autoregulatory responses to reductions in arterial blood pressure. Additionally, mild bTBI resulted in significantly impaired working memory 2 weeks post-bTBI, and led to increased numbers of FJC-positive cells throughout the cortex of the rat brain, with the greater quantity located among areas overlapping the frontal and parietal/temporal regions. We also observed significant reductions in blast-induced increases in MAP and significant reductions in cerebral vascular resistance after treatment with the ONOO^−^ scavenger PenME, suggesting that blast-induced cerebral vascular dysfunction may be, in part, a result of the action of oxidants such as ONOO^−^.

These studies were conducted using an ABS, a new shock tube that has been used in several recent bTBI studies.^55,83–85^ The ABS is designed to circumvent some of the problems associated with experimental blast research. The ABS is equipped with a reflected wave suppressor that prevents reflection of pressure waves back into the specimen chamber that are produced when the primary blast wave interacts with either the closed or the open end of the tube.^86^ The divergent area driver chamber and expansion section of the ABS are designed to produce a more accurate shock waveform.^85^ A potential limitation of shock tubes is the impact of fragments produced by the rupture of membranes.^87^ Acetate membranes, which are utilized in other ABS devices, produce true Friedlander-type overpressure waves, but the fragments produced may act as projectiles, resulting in a combined insult of primary blast wave exposure plus secondary impacts from acetate fragments. In contrast, Mylar ruptures without producing fragments, making it better suited for studies of the effects of shock-wave exposure. Although the incomplete rupture of Mylar membranes may alter the shape of the overpressure wave in some studies,^87^ the pressure wave generated by the ABS using Mylar membranes in the present study closely approximated that of an idealized Friedlander wave.

Although bTBI was associated with (1) impaired cerebral perfusion and dilator responses to reduced intravascular pressures, (2) neuronal injury, and (3) cognitive dysfunction, the mild shock-wave levels used in these studies resulted in durations of suppression of the righting reflex (<1 min) only slightly higher than those in sham-injured rats. In experimental studies of bTBI, as in non-blast TBI, there is some overlap in overpressure levels considered to result in “mild” or “moderate” bTBI.^88^ Therefore, we based our definition of mild bTBI on a duration of suppression of the righting reflex of ≤7 min; our definition of moderate bTBI on a duration of suppression of the righting reflex of between 8 and 14 min; and our definition of severe bTBI on a duration of suppression of the righting reflex of >14 min.^38,88–91^ The righting reflex is a brainstem reflex that is widely used in studies of the effects of anesthetics^92,93^ or brain injury.^94–96^ Duration of suppression of the righting reflex is considered to be analogous to duration of loss of consciousness, one of the most consistent criteria for the definition of mild TBI in humans.^88^ However, whereas most definitions of mild TBI in humans assume normal imaging, mild TBI in experimental animals may be associated with significant neuronal injury.^88^

The pathobiology of primary bTBI consists of a complex set of systemic, cerebral, and cerebral vascular events that begin at blast exposure and likely continue for hours to days or weeks afterwards.^3,10,13,14^ Although there have been many studies on the histopathological and behavioral effects of blast exposure,^2,13,37,38,53,57,97–108^ there have been fewer dedicated to the cerebral vascular effects of bTBI. Previous research suggests that bTBI is associated with cerebral vascular injury.^40^ Pun and colleagues^109^ observed histological evidence of narrowing of cerebral vessels in rats 1 and 4 days after low level (7.1 or 11.3 psi), whole body, explosive blast exposure. Gama Sosa and colleagues^42^ reported abnormal collagen IV and laminin staining in the primary visual cortex in rats 24 h after shock-wave exposure (10.8 psi) as well as varying degrees of microvascular pathology (e.g., focal and/or shear-related lesions, focal hemorrhage, and isolated intraparenchymal microhemorrhages) that persisted for up to 10 months post-blast. Kwon and colleagues^110^ and Kovesdi and colleagues^111^ reported elevated vascular endothelial growth factor levels more than 2 months after shock-wave injury (20.6 psi) in rats. Although these results indicate that bTBI results in cerebral vascular injury that may persist for months post-injury, the question of whether bTBI is associated with acute (< 24 h) alterations in cerebral perfusion and cerebral vascular reactivity is unexplored.

Our results that relative cerebral perfusion was reduced in the bTBI group indicate that mild bTBI contributed to significant cerebral hypoperfusion that started within 5 min of and persisted for at least 2 h after ABS injury. Bir and colleagues,^33^ using MRI methods to measure relative cerebral perfusion, reported that shock-wave exposure (13–27 psi) resulted in significantly reduced relative perfusion from 24 to 72 h post-injury in rats. In the present study, as in that of Bir and colleagues,^33^ the rats were anesthetized with isoflurane, a volatile anesthetic^112,113^ that increases CBF in a concentration-dependent manner.^114,115^ Considering the vasodilatory properties of isoflurane,^116–123^ it is likely that the blast-induced reductions in cerebral perfusion would have been greater in the absence of the vasodilatory effects of isoflurane.

Our results also indicated that compensatory dilator responses to reduced intravascular pressure in MCA segments were significantly reduced 30 and 60 min after mild bTBI. The myogenic vascular response, a major mechanism contributing to CBF autoregulation and reductions in arterial blood pressure and first described by Bayliss,^124^ is characterized by vasoconstriction if perfusion pressure increases, and by vasodilatation if perfusion pressure decreases.^125,126^ Impairment of endothelium-dependent dilator responses to acetylcholine in isolated basilar arteries harvested from rats subjected to single or repeated shock-wave exposure (30 psi) was reported by Toklu and colleagues.^34^ These investigators also reported that constrictor responses to endothelin-1 in basilar arterial segments were enhanced by shock-wave exposure. Increased endothelin-dependent vasoconstriction after blast would be consistent with our observations that cerebral vascular resistance was significantly increased by bTBI. Alford and colleagues^81^ reported that blast exposure *in vitro* resulted in increased sensitivity to the constrictor effects of endothelin-1 within 1 h of simulated blast exposure (high velocity uniaxial stretch) in a highly-aligned monolayer of vascular smooth muscle cells on an elastic substrate. Further, 24 h after high velocity stretch injury, the vascular smooth muscle cells exhibited prolonged hyperconstriction dependent on the force of the stretch. Bauman and colleagues^37^ reported cerebral arterial vasospasm after blast exposure in swine *in vivo*. Armonda and colleagues^82^ reported cerebral vasospasm in 48% of patients with severe blast-related trauma. Although cerebral vasospasm often was observed after bTBI in combat casualties and is common after subarachnoid hemorrhage, Armonda and colleagues^82^ observed vasospasm in bTBI patients even in the absence of subarachnoid hemorrhage. Together, our results and those of Toklu and colleagues,^34^ Alford and colleagues,^81^ and Armonda et al.^82^ suggest that blast exposure may contribute to cerebral arterial vasospasm by impairing cerebral vascular dilator mechanisms.

Impact (i.e,. non-blast) TBI markedly increases the mortality and morbidity of post-traumatic insults such as hemorrhagic hypotension,^127–129^ in part because traumatic cerebral vascular injury impairs or abolishes compensatory cerebral vasodilation to reduced intravascular pressure.^17,25,55,130^ Although our study did not include any groups exposed to hemorrhagic hypotension, our results and those of Toklu and colleagues^34^ indicate that bTBI-impaired cerebral vasodilatory responses from blast exposure, like impact TBI, may increase vulnerability to post-blast hemorrhagic hypotension. This hypothesis is supported by the work of Long and colleagues,^13^ who observed that survival rates were significantly lower in rats subjected to hemorrhagic hypotension after blast injury than in sham-injured rats. Unfortunately, the likelihood that combatants exposed to bTBI also sustain post-traumatic hemorrhagic hypotension is high. Hemorrhage is the leading cause of death in combat, including those killed instantly (killed in action) and those who died after transport to a medical facility (died of wounds).^131^ Chambers and colleagues^132^ reported that 90% of patients treated by the United States Marine Forward Resuscitation Surgical System (FRSS) during Operation Iraqi Freedom sustained penetrating injuries and, therefore, some degree of hemorrhage. More than 80% of the most critically ill patients treated by the FRSS presented in class three or four hemorrhagic shock, and 40% of patients who required treatment during transport to higher echelons of care were treated for systemic arterial hypotension.^132^ Nelson and colleagues^133^ observed that 100% of blast-injured patients with persistent arterial hypotension (systolic blood pressure <90) died, whereas all of those without hypotension survived their injuries. Although the results of Nelson and colleagues^133^ suggest that hemorrhagic hypotension after blast injury is associated with higher mortality, it was a small study (*n* = 14,) and it is possible that mortality was high in the patients with hypotension because they were more seriously injured. Although these results are inconclusive, they are consistent with a hypothesis that bTBI, like non-blast TBI, renders patients more vulnerable to post-blast hemorrhagic hypotension.

**FIG. 8.**
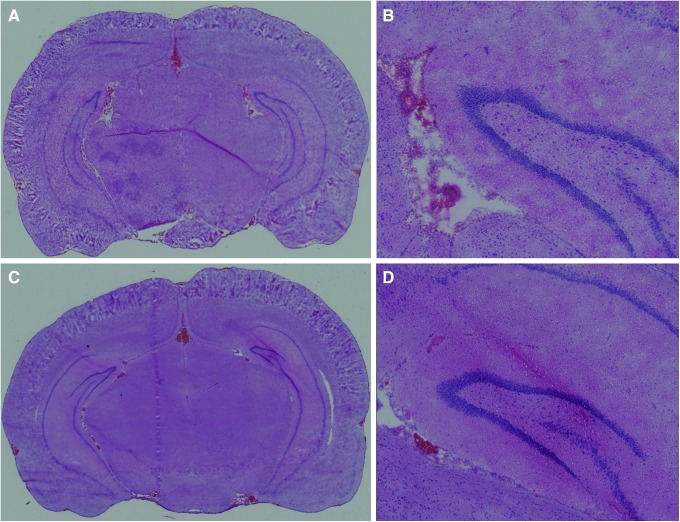
Hematoxylin and eosin staining of coronal sections at bregma ±0.3 24 h after (**A**) sham injury and (**B**) blast-induced traumatic brain injury (bTBI). No abnormalities were observed in either section. Higher magnification images of the (**C**) sham and (**D**) bTBI sections of **A** and **B**, respectively, revealed intraventricular blood near the choroid plexus in both brains, but no histological abnormalities in or around the cortex and hippocampal regions of either section. Images **A** and **B** were taken at 8 × magnification; images **C** and **D** were taken at 20× magnification. Color image is available online at www.liebertpub.com/neu

Mild bTBI resulted in significant increases in the numbers of FJC-stained cells in the frontal and parietal/temporal regions, but the numbers were relatively small and we did not observe consistent increases in FJC staining in the hippocampus after bTBI. The selective nature of the FJC staining and the absence of histopathological evidence of injury in hematoxylin and eosin-stained sections (Fig. 8) from the brains of rats subjected to mild bTBI are consistent with the mild level of bTBI used in these studies. In comparison with other studies in which FJ staining was used to quantify neuronal injury after what appear to be comparable bTBI levels, we observed fewer FJ-stained cells.

Sajja and colleagues^134^ reported 116.2 ± 11.3 and 191.8 ± 68.4 FJB-stained hippocampal neurons/mm^2^ 24 and 48 h after 117 kPa (17 psi) shock tube injury, respectively, in rats. In another study, Sajja and colleagues^83^ observed ∼150–210 FJB-positive neurons (estimated from Sajja and colleagues^83^ Figure 5) in the prefrontal cortex of rats 3–168 h after 117 kPa (17 psi) shock tube injury. In a third study, Sajja and colleagues^84^ counted hundreds to thousands of FJB-stained cells in the hippocampus, amygdala, prefrontal cortex, and nucleus accumbens in rats 1–3 months after 117 kPa (17 psi shock tube injury [see Sajja and colleagues^84^ Table 1]). Because we counted FJC-stained cells throughout the cerebral hemisphere whereas Sajja and colleagues counted cells in specific brain regions (e.g., hippocampus, prefrontal cortex), it is clear that we observed more limited neuronal injury; however, the reasons for the differences are uncertain. Although the levels of shock-wave overpressure appeared similar between our study (peak overpressure ∼20 psi) and those of Sajja and colleagues (∼17 psi), the overpressure duration was greater in two of the studies by Sajja and colleagues. In two studies by Sajja and colleagues,^84,134^ peak overpressure duration was 7.5 msec in contrast to 3.5 msec in our study. Assuming that the shapes of overpressure versus time curves in our studies and those of Sajja and colleagues were similar, the longer duration in the studies of Sajja and colleagues would have resulted in higher levels of blast impulse, the area under the overpressure curve. Because impulse is the primary determinant of blast severity,^135^ it is likely that blast injury severity was higher in the studies by Sajja and colleagues. Additional evidence that blast severity was higher in the studies of Sajja and colleagues is the difference in the temporal pattern of neuronal injury. Sajja and colleagues reported more FJ-positive neurons in the rat hippocampus 48 versus 24 h after bTBI,^134^ increases in the numbers of FJ-positive neurons in the prefrontal cortices between 3 and 168 h post-bTBI,^83^ and increases in the numbers of FJ-positive cells between 1 and 3 months after blast in the hippocampus, amygdala, prefrontal cortex, and nucleus accumbens.^84^

In contrast, we observed decreases in the numbers of FJ-positive cells between 24 and 48 h post-bTBI, perhaps because our mild blast injury permitted some degree of neuronal recovery whereas the more severe injury used by Sajja and colleagues resulted in ongoing neuronal injury that continued or worsened for days to months after bTBI. However, because we based our assessments of blast severity on the duration of suppression of the righting reflex, which was not measured in the studies by Sajja and colleagues, definitive comparisons of injury severities between this study and those of Sajja and colleagues are not possible.

Although most studies of the histopathological effects of bTBI used shock tubes, Kuehn and colleagues^136^ observed increases in the numbers of FJ-positive neurons in the cerebellum and hippocampus of rats subjected to severe blast exposure (427–517 kPa, 62–75 psi) in a blast tube powered by nail gun blank cartridges. In addition to neuronal injury assessed using FJ staining, Cho and colleagues^55^ observed reduced neuronal nuclear antigen immunoreactivity in the hippocampus from 24 h to 2 weeks post-blast using an ABS shock tube similar to ours, and comparable shock-wave amplitudes (19 psi, 129 kPa). As neuronal nuclear antigen is a protein expressed by mature neurons, the results of Cho and colleagues^55^ indicated that bTBI was associated with the loss of hippocampal neurons that continued for at least 2 weeks post-blast. Together, these reports and our results indicate that mild primary blast/shock-wave exposure results in significant neuronal injury that lasts at least 6 months post-blast.

In addition to cerebral vascular and cellular injury, we observed significant impairment in working memory. The differences between the time to the goal platform in trials 1 and 2 of the MWM was significantly greater in the sham than in the bTBI group on days 11–15 after blast or sham blast. Because rats with normal working memory will find the goal platform more rapidly during the second trial of each pair, the differences between the trial pairs would be larger. Similar latencies between the first and second trials of each pair are indicative of working memory dysfunction, as we observed. These results are consistent with previous reports of blast-induced memory deficits. Sajja and colleagues^83^ and Cho and colleagues^55^ reported working memory deficits (novel object recognition) 1–2 weeks after 117 kPa shock-wave exposure. Sajja and colleagues^84^ observed deficits in working memory (novel object recognition) that persisted for at least 3 months after 117 kPa shock-wave exposure. Spatial memory deficits (Barnes maze) were observed in rats 2 weeks^130^ or 2 months^104^ after 138 kPa (20 psi) or 142 kPa (21 psi) shock-wave exposure, respectively. These results indicate that mild bTBI results in working and spatial memory deficits that may persist as long as 3 months after blast exposure.

Several studies strongly suggest that one of the principal reactive oxygen species involved in the secondary injury process of TBI pathophysiology and neurodegeneration is the nitrogenous ONOO^−^.^48,49,137–139^ ONOO^−^-mediated damage can be reduced, however, through the administration of reactive oxygen species scavengers. Scavenging compounds have previously shown neuroprotective properties in various models of experimental TBI.^140–144^ Althaus and colleagues^75^ investigated cysteine analogs as scavengers of ONOO^−^ and observed their efficacy from nitric oxide toxicity. Of these compounds, there is direct chemical evidence that Pen (L-*β*,*β*-dimethylcysteine) is a stoichiometric (1:1) scavenger of peroxynitrous acid^48^ and ONOO^−^.^48,75,145^ Althaus and colleagues^75^ observed that Pen directly reacts with ONOO^−^ to form a single *S*-nitro-L-Pen adduct, whereas Hall and colleagues^48^ reported that Pen and PenME (methyl [2R]-2-amino-3-methyl-3-sulfanylbutanoate) improved outcome and neurological recovery^48,49^ after TBI in a dose-dependent manner. Whereas Pen has limited blood–brain barrier permeability and acts intravascularly, PenME is a lipophilic, blood–brain barrier-permeable free radical scavenger^48^ that can act extravascularly. Hall and colleagues^48^ reported that both Pen and PenME improved early neurological outcome (grip test) after TBI in mice, suggesting that intravascular as well as extravascular scavenging of ONOO^−^ contributed to the therapeutic effects of Pen. Moreover, bTBI can result in blood–brain barrier breakdown leading to increased blood–brain barrier permeability,^38,39^ thereby increasing the access of substances that normally would not traverse the blood–brain barrier. We tested the PenME compound as our experimental free radical scavenger in order to determine the cerebral vascular effects of ONOO^−^ scavenging after bTBI as measured by relative cerebral perfusion and mean arterial pressure in rats treated with PenME after blast injury. Our results of a trend toward increases in cerebral perfusion and significant reductions in cerebral vascular resistance after treatment with PenME suggest that blast-induced cerebral vascular dysfunction is caused, in part, by the actions of ONOO^−^.

The manner in which blast energy is transmitted into the brain remains controversial.^13,14,98,146–152^ One of several theorized mechanisms of primary blast injury is the transfer of kinetic blast energy to the cerebral vasculature and brain via the great vessels of the thorax.^14,98,146,147,151^ In studies of intracranial pressure oscillations in an unprotected, whole-body shock-wave-exposed Rhesus monkey^153^ and in a swine outfitted with a lead-and-foam-lined vest that covered the chest and upper abdomen,^37^ significant increases in intraparenchymal and intravascular pressure pulses were observed. Experimental blast studies in rodents demonstrated that protecting the torso virtually eliminated axonopathy and fiber degeneration,^13^ whereas the use of a plexiglass covering around the torso of blast-injured mice abolished axonal nerve cell damage compared with unshielded mice who sustained up to 80% axonal damage.^152^ In contrast, in ferrets^154^ and rabbits^155^ with thoracic and abdominal protection, apnea, meningeal bleeding (ferrets), and multifocal subdural and subarachnoid hemorrhages (rabbits) as well as fatalities were observed. In a rat model in which blast overpressures were delivered exclusively to the head through direct cranial blast injury,^136^ sublethal injury resulted in apnea, subarachnoid hemorrhages in the path of the blast wave, abnormal immunoglobulin immunolabeling, cleaved caspase-3 and β-amyloid precursor protein, FJC staining in brain regions not overlapping the subarachnoid hemorrhages, and abnormalities on the behavioral rotarod task. These results of blast-induced brain injury in the presence of thoracic protection indicate that primary blast exposure to the head in the absence of thoracic injury is sufficient to produce significant brain injury. We used head-only blast exposure to determine blast effects to the head alone, excluding the possibility of indirect brain injury through thoracic transmission of the blast wave.

Although our results are consistent with reduced cerebral blood perfusion leading to acute cellular injury/degeneration and impaired working memory and/or cognitive dysfunction, we cannot definitively establish a correlation that these results of blast-induced reductions in cerebral perfusion contributed to either the neuronal injury seen and quantified throughout the brain or the observed impaired working memory performance. We do not definitely know how long the reduced cerebral perfusion observed lasts past the 2 h time point examined in our studies. Additionally, this study was not designed to show a cause and effect nature from one experiment to the next, as different groups of animals were studied for each set of experiments and at different time points. FJC-positive cells were counted throughout the entire brain, whereas relative cerebral perfusion was measured only at one location in the cerebral cortex. Working memory performance is particularly sensitive to hippocampal injury, but hippocampal perfusion was not measured. Rather, these studies were designed to better characterize the effects of mild bTBI on righting reflex suppression, cerebral dilator responses to reduced intravascular pressure, cerebral perfusion, mean arterial pressure, cerebral vascular resistance, acute cellular injury/degeneration, and vestibulomotor and cognitive dysfunction.

## Conclusion

Our results indicate that mild bTBI levels associated with statistically significant but small increases in the duration of suppression of righting reflexes produced significant reductions in relative cerebral perfusion and significant increases in cerebral vascular resistance. Additionally, these studies indicated that mild bTBI resulted in significant increases in the numbers of FJC-positive cells throughout the brain and significant impairments in working memory performance. Our results that cerebral vasodilator responses to reduced intravascular pressure in MCA segments were impaired by mild bTBI suggest that blast exposure may result in increased vulnerability to hemorrhagic hypotension. Finally, our results showing that PenME significantly reduced blast-induced increases in cerebral vascular resistance suggests that ONOO^−^ contributes to the impairment of cerebral vascular function after bTBI.
